# THE ASSOCIATION BETWEEN ACCESS TO MEDICAL CARE AND RESIDENT OUTCOMES IN NURSING HOMES

**DOI:** 10.1093/geroni/igac059.2591

**Published:** 2022-12-20

**Authors:** Krittika Bali

**Affiliations:** University of Alberta, Edmonton, Alberta, Canada

## Abstract

The nursing home population is vulnerable and medically complex, yet little is known about models of medical service provision and associated quality outcomes. The goal of this study is to examine the association between physician (MD) and nurse practitioner (NP) accessibility and practice sensitive outcomes. This project used data from the Translating Research in Elder Care (TREC) longitudinal study and the routinely collected Resident Assessment Instrument – Minimum Data Set version 2.0 (RAI-MDS 2.0) to test the association between the availability of MDs and NPs in nursing homes (NH) and clinically-relevant resident outcomes of antipsychotic medication (APM) use without indication of psychosis, physical restraint use, hospitalization and emergency department (ED) transfers, and polypharmacy. Eight models were created using logistic regression to test the association between the access measures of daily presence of MD or NP on unit and MDs being involved in care planning and each of the four resident outcomes. The sample consisted of 10,888 residents across 320 units in 92 facilities. Staff from 277 (86%) units reported an MD or NP visited daily and 318 (99%) units reported that the MD or NP could be reached when needed. Following adjustment for multiple confounding variables, there were no associations between either measure of access and any of the resident outcomes. Although we did not find any associations between our measures of access and resident outcomes, additional research which more directly measures physician and NP activities in the NH is required.

